# Quantitative trait loci for partial resistance to *Pseudomonas syringae* pv. *maculicola* in *Arabidopsis thaliana*

**DOI:** 10.1111/mpp.12043

**Published:** 2013-06-03

**Authors:** Jenni C. Rant, Lia S. Arraiano, Matthieu Chabannes, James K. M. Brown

**Affiliations:** ^1^ John Innes Centre Colney Norwich NR4 7UH UK; ^2^ Present address: Vilmorin SA Centre de Recherche de La Costière 30210 Ledenon France; ^3^ Present address: CIRAD UMR BGPI F‐34098 Montpellier France

## Abstract

Segregation of partial resistance to *Pseudomonas syringae* pv. *maculicola* (*Psm*) ES4326 was studied in the recombinant inbred population created from accessions (ecotypes) Columbia (Col‐4), the more susceptible parent, and Landsberg (Ler‐0). Plants were spray inoculated with lux‐transformed bacteria in experiments to measure susceptibility. The amount of disease produced on a range of Col × Ler lines by spray inoculation was highly correlated with that produced by pressure infiltration of bacteria into the apoplast. Quantitative trait locus (QTL) analysis identified four loci that contributed to partial resistance: *QRps.JIC‐1.1*, *QRps.JIC‐2.1*, *QRps.JIC‐3.1* and *QRps.JIC‐5.1* on chromosomes 1, 2, 3 and 5, respectively*. QRps.JIC‐3.1*, located 8.45 cM from the top of the consensus genetic map of chromosome 3, had a large, approximately additive effect on partial resistance, explaining 50% of the genetic variation in this population. Fine mapping narrowed the region within which this QTL was located to 62 genes. A list of candidate genes included several major classes of resistance gene.

## Introduction

Partial resistance in plants is a form of defence which has an incomplete effect against all genotypes of a pathogen. It contrasts with the resistance conferred by resistance (*R*) genes controlling gene‐for‐gene interactions, which provide near‐complete defence, but only against avirulent pathogen genotypes. There are many, diverse kinds of interaction between plants and parasites, and there is no single genetic or phenotypic model for partial resistance (Johnson, 1984). It can be defined empirically as a form of defence which increases the latent period, slows disease progress or reduces the severity of symptoms despite an essentially compatible interaction and, in so doing, decreases the rate of epidemic development. Partial resistance is generally not specific to different genotypes of one pathogen species, although it can vary in its quantitative effectiveness against different, related pathogens (Gonzalez *et al*., 2012). This type of host defence therefore provides durable resistance to many crop diseases and has widespread importance in plant breeding (Johnson, 1984; St Clair, 2010; Stuthman *et al*., 2007).

The genetic systems controlling partial resistance to disease are as diverse as the phenotypes. There is substantial quantitative variation in many plants, including crops and models, such as *Arabidopsis*. In some cases, a few genes account for a large proportion of the variation, as with the genes *Lr34* (Krattinger *et al*., 2009) and *Lr46* (Singh *et al*., 1998) for resistance to the rusts of wheat. In most cases, however, partial resistance is polygenic, with many genes each having a small effect. Some genes for partial resistance have been mapped as quantitative trait loci (QTLs) but, in other cases, a polygenic system has been inferred, because a substantial fraction of genetic variation is not explained by genes with effects sufficiently large to exceed a threshold for detection. This is the case even for important sources of resistance to major diseases, such as that of the wheat cultivar Arina to *Septoria tritici* blotch (Chartrain *et al*., 2004). The quantitative nature of resistance and the comparatively small effects of many genes effective against significant diseases mean that, in contrast to major *R* genes, few genes for partial resistance in crop plants have been isolated and characterized (St Clair, 2010; also see Cook *et al*., 2012).

One component of partial resistance might be basal resistance, in which pathogen‐associated molecular patterns (PAMPs) act as elicitors, triggering the perception of biotic threats and inducing complete or partial defence. Transmembrane pattern recognition receptors (PRRs) that respond to PAMPs, such as bacterial flagellin or fungal chitin, elicit the up‐regulation of diverse genes involved in basal disease resistance in *Arabidopsis* (Wan *et al*., 2008; Zipfel *et al*., 2004). The relationship between partial resistance in crops and basal resistance, however, has not yet been elucidated. The identification of genes which modify partial resistance without preconceptions about their function or homology should lead to a fuller understanding of this type of defence, including the interaction of PAMP‐triggered immunity with other components of partial resistance.

A significant challenge in the study of partial resistance is that it must be measured quantitatively, in contrast to major *R* genes which can often be scored qualitatively as present or absent. The development of rapid, efficient methods of studying partial resistance will advance research on this important trait. The study of the mechanisms of partial resistance may be further accelerated by the use of a model host, such as *Arabidopsis*, in which genetic analysis is relatively rapid, information can be transferred readily from genetic to physical maps and new traits can be related to a wealth of knowledge about the plant's genetics, physiology and molecular biology.

Here, we report a QTL analysis of resistance to the bacterial pathogen *Pseudomonas syringae* pv. *maculicola* (*Psm*) ES4326. There is a gene‐for‐gene relationship in this host–pathogen interaction, but there is also quantitative variation in the responses of accessions lacking a gene‐for‐gene *R* gene effective against the bacterium (Kover and Schaal, 2002). Resistance to this bacterium segregated in a population of *Arabidopsis* recombinant inbred lines (RILs) bred from the cross Columbia‐4 × Landsberg *erecta*‐0 (Col × Ler; Lister and Dean, 1993), which has been used to analyse the genetics of many traits. The development of a lux‐tagged strain of *Psm* ES4326 enabled efficient recording of the growth of bacteria *in planta*, using a luminometer that measured relative light units (RLU) emitted by the lux tag (Fan *et al*., 2008). A list was compiled of likely candidate genes for each of the four QTLs identified, including several from significant known classes of resistance gene; at least three of the QTLs are reported here for the first time.

## Results

### Disease symptoms and partial resistance

**Figure 1:**
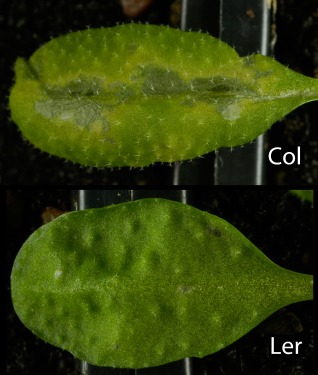
*Arabidopsis thaliana* leaves 3 days after spray inoculation with *Pseudomonas syringae* pv. *maculicola* ES4326 + lux tag at 5 × 10^7^ colony‐forming units (cfu)/mL. Columbia (Col) showed water‐soaked patches, which are typical disease symptoms, whereas Landsberg (Ler) showed only a few signs of infection.

Of the Col × Ler population of 300 RILs, 98 were used to generate data for disease caused by *Psm* on *Arabidopsis*. ES4326, a virulent strain of *Psm* lacking known effective avirulence (*AVR*) genes, was applied to plants by spraying to mimic the natural process of splash‐borne infection. Columbia has been shown previously to be susceptible to *Psm* ES4326 applied by infiltration, with water‐soaked patches visible 2 days after inoculation (dai), and chlorotic and necrotic patches by 3 dai (Dong *et al*., 1991). Symptoms in Columbia following spray inoculation replicated those found by Dong *et al*. (1991), but Landsberg was more resistant than Columbia, showing few symptoms by 3 dai (Fig. 1). The RILs therefore have Columbia as the more susceptible parent and Landsberg as the more resistant parent.

**Figure 2:**
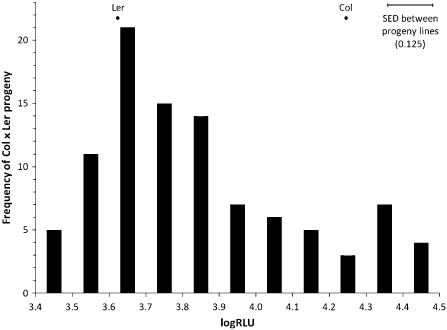
Frequency distribution of mean pathogen scores of Columbia × Landsberg recombinant inbred lines expressed as log_10_ relative light units of luminescence emitted by the lux tag (logRLU) in *Pseudomonas syringae* pv. *maculicola* ES4326 inoculated by spraying at 5 × 10^7^ colony‐forming units (cfu)/mL. Mean scores for parents: Landsberg *erecta* (Ler), 3.623; Columbia (Col), 4.245. Lower scores imply greater partial resistance. Standard error of difference (SED) between parents, 0.044; SED between parents and progeny lines, 0.089.

The RILs displayed a range of susceptibility to *Psm* ES4326, measured as the predicted mean score of bacterial infection, with lower scores indicating greater resistance (Fig. 2). Columbia was the more susceptible parent, with a mean disease score of 4.245 logRLU (log_10_ RLU), and Landsberg was the more resistant parent, with a mean disease score of 3.623 logRLU; the standard error of the predicted means for parents was 0.031 logRLU. There was no clear segregation into distinct classes of more resistant and more susceptible individuals (Fig. 2), suggesting that partial resistance was controlled by more than one gene. There was transgressive segregation of pathogen numbers in infected plants, implying that both parents have genes at different loci associated with partial resistance; at the extremes, RIL N1925 had a mean of 3.418 logRLU and N1970 had a mean of 4.498 logRLU. This surpassed the range of the parents [Fisher's protected least‐significant difference (*P <* 0.05) between parents and progeny lines: 0.175].

**Figure 3:**
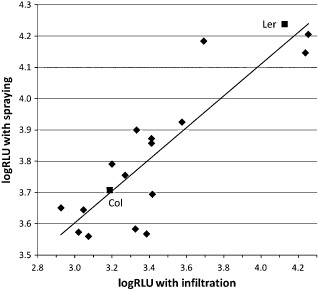
Disease scores of the parents and 16 recombinant inbred lines (RILs) of the Columbia × Landsberg RIL population expressed as log_10_ relative light units of luminescence emitted by the lux tag (logRLU) in *Pseudomonas syringae* pv. *maculicola* ES4326. The pathogen was inoculated by infiltration at 10^5^ colony‐forming units (cfu)/mL or by spraying (data from Fig. 2). Correlation coefficient (*r*) = 0.88. Regression equation: *S* = 0.51*F* + 2.81, where *S* and *F* are the predicted mean logRLU scores from spraying and infiltration, respectively. Col, Columbia; Ler, Landsberg.

A subset of 16 RILs plus the two parental accessions were inoculated by pressure infiltration, a method commonly used in research on *Pseudomonas* of *Arabidopsis*, so that the susceptibility of plants to *Psm* applied by this method could be compared with the results obtained by spraying. The 16 RILs covered all possible combinations of alleles of the four QTLs (see next section). The responses of the lines to *Psm* inoculated by the two methods were highly correlated (*r* = 0.88, *P <* 0.001; Fig. 3).

### QTL mapping

**Figure 4:**
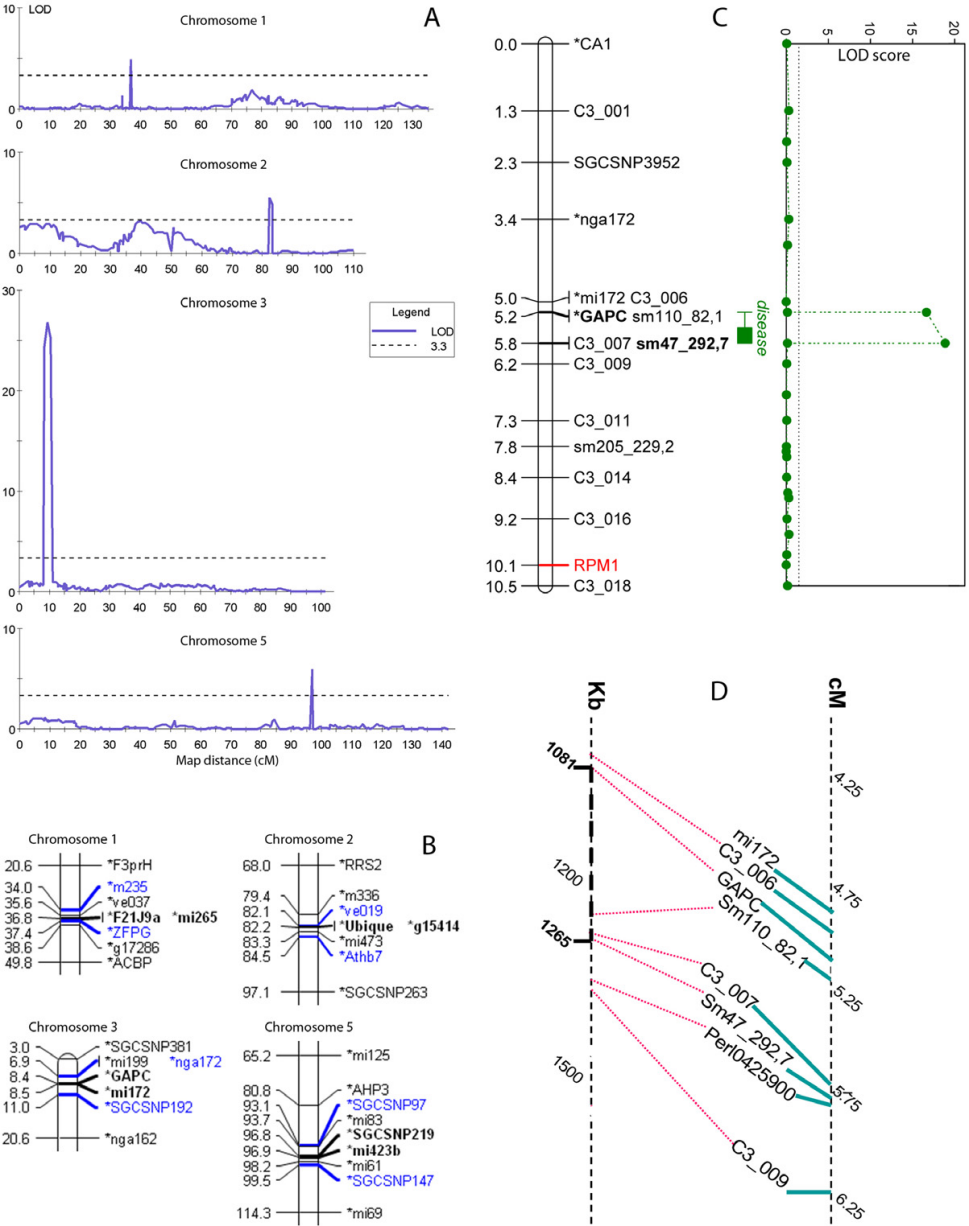
(A) Charts of quantitative trait loci (QTLs) in the Columbia × Landsberg (Col × Ler) population of recombinant inbred lines for partial resistance to a virulent strain of *Pseudomonas syringae* pv. m*aculicola*, ES4326. Map distances on the abscissa are plotted by reference to Singer *et al*. (2006). The broken line on the ordinate at logarithm of the likelihood ratio (LOD) = 3.3 indicates the *P <* 0.05 threshold of LOD for the identification of a QTL. (B) Markers in bold indicate the maximum likelihood positions of QTLs, whereas those in blue were used to delimit QTL regions. Maps of markers around the four QTLs. (C) Fine mapping of QTL *QR* *ps.JIC‐3.1* at the top of chromosome 3, accounting for 50% of the partial variation. Map distances (cM) are shown on the left with the LOD profile in green on the right. Markers in bold delimit the QTLs and the locus of *RPM1* is indicated in red. (D) Positions of chromosome 3 markers on genetic and physical maps.

Predicted mean logRLU scores were used to search for QTLs associated with partial resistance, to estimate the number of genes that contributed to partial resistance, their relative importance and their locations. Multiple QTL Model (MQM) mapping was performed using 1290 of a possible 1357 markers from the Nottingham Arabidopsis Stock Centre (NASC) website. Four QTLs with a significant association with partial resistance [*P <* 0.05; logarithm of the likelihood ratio (LOD) > 3.3; Fig. 4A,B] were identified (Table 1). A second round of mapping was performed with another, smaller set of 676 markers (Singer *et al*., 2006) which had complete genotype scores for all the RILs; the NASC marker genotype scores are incomplete, but cover a larger proportion of the five chromosomes.

**Table 1:** Quantitative trait loci (QTLs) for partial resistance to *Pseudomonas syringae* pv. *maculicola* ES4326 in the Columbia × Landsberg (Col × Ler) recombinant inbred population, with locations (cM from the top of the consensus genetic map), logarithm of the likelihood ratio (LOD), percentage variation explained, additive effect of the allele in Columbia on increasing disease (logRLU score), delimiting loci and the number of genes between these loci

Chromosome and QTL name	Position (cM)	LOD	% variation explained	Col allele effect on disease score	Interval size (kbp)	Delimiting loci	Total genes in interval
1: *QRps.JIC‐1.1*	36.81	4.8	5.5	+0.07	370	At1g23450–At1g24530	150
2: *QRps.JIC‐2.1*	82.21	5.5	6.4	−0.07	496	At2g45090–At2g46510	203
3: *QRps.JIC‐3.1*	8.45	26.7	50.3	+0.21	221	At3g04120–At3g04660	62
5: *QRps.JIC‐5.1*	96.87	5.9	6.9	+0.08	819	At5g43755–At5g45400	201

Alignment of the graphs of log‐likelihood against the genetic maps indicated that, as expected, the QTLs mapped in the same positions with both sets of markers. The most important QTL was located on the top arm of chromosome 3, approximately 8 cM from the telomere. It had a LOD score of 26.8 and accounted for 50.3% of the genetic variation between the mean logRLU scores of the lines. The Col allele at this locus was associated with greater susceptibility and the Ler allele with increased resistance. The remaining three QTLs on chromosomes 1, 2 and 5 had LOD scores of 4.9, 5.5 and 5.9, respectively, and together explained 18.8% of the variation in the mean scores of the RILs, with a smaller effect on logRLU than the QTL on chromosome 3. There is no established system for naming QTLs in *Arabidopsis*. We used the method employed in wheat genetics, in which QTL names consist of the prefix Q followed by a three‐letter descriptor of the phenotype, an indicator for the laboratory, the chromosome number and a serial number. The QTLs were named *QRps.JIC‐1.1*, *QRps.JIC‐2.1*, *QRps.JIC‐3.1* and *QRps.JIC‐5.1*, representing QTLs for Resistance to *Pseudomonas syringae* identified at the John Innes Centre. Together, the four QTLs represent 69.1% of the total genetic variation in the mean partial resistance of the lines (Table 1). Further analysis of mean disease scores for all the lines with genotype data did not detect any significant epistasis between the QTLs (not shown).

**Figure 5:**
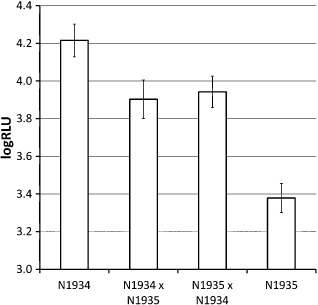
Disease scores of F1 plants and recombinant inbred lines (RILs) chosen for crossing, which differed for markers flanking the quantitative trait locus (QTL) *QRps.JIC‐3.1*, but were isogenic for the other three QTLs. Data are predicted mean disease scores expressed as log_10_ relative light units of luminescence emitted by the lux tag (logRLU) in *Pseudomonas syringae* pv. *maculicola* (*Psm*) ES4326 inoculated by infiltration with a bacterial suspension at 1 × 10^5^ colony‐forming units (cfu)/mL. Bars indicate one standard error above and below the predicted mean. The fact that the mean scores for the F1 lines are intermediate between those of the crossed RILs implies that the *QRps.JIC‐3.1* allele in Landsberg *erecta* and line N1935 has an approximately additive effect on resistance to *Psm*.

Lines N1934 and N1935 were identified as differing by their genotypes of markers flanking the QTL on chromosome 3, but not in markers linked to the other QTLs. Reciprocal crosses were made between these lines and the F1 plants were tested for their responses to *Psm* alongside the crossed RILs. Plants were inoculated by infiltration rather than spraying, so that leaf material could be recovered for genotyping. The logRLU scores of the F1 plants were intermediate between those of the crossed RILs (Fig. 5), implying that the effect of *QRps.JIC‐3.1* on partial resistance to *Psm* is approximately additive. There was no significant difference between the mean scores of the reciprocal F1 lines (*P* = 0.7), so there was no evidence for a maternal effect on partial resistance.

### Fine mapping of *QRps.JIC‐3.1*

New markers were designed within the QTL region on chromosome 3 defined by the NASC markers, whereas additional markers (Singer *et al*., 2006) were added to enrich the genetic map in order to narrow the interval containing this QTL, much the most potent of the four detected (Fig. 4C). The physical positions documented on The Arabidopsis Information Resource (TAIR) website for the delimiting markers, GAPC and Sm47_292,7, were aligned with the genetic map (Fig. 4D), enabling the use of the TAIR website's seqviewer facility, which returns an annotated list of genes in any specified interval in the *Arabidopsis* genome. Sixty‐two predicted genes were located between the markers flanking the QTL on the revised map (loci At3g04120 to At3g04660).

### Candidate genes

Together, the four QTL regions, identified from mapping only with the Singer markers and following fine mapping of *QRps.JIC‐3.1*, included a total of 616 predicted genes identified using the TAIR seqviewer (Table 1). The Singer marker set was used in this analysis because the QTL peaks were located in the same positions whether the NASC or Singer markers were used in mapping, but there are no missing genotype scores in the Singer data. All 616 genes were analysed for the presence of polymorphisms between the sequences of the parental accessions, Ler and Col, using the GBrowse Viewer (http://gbrowse.weigelworld.org/cgi‐bin/gbrowse/polymorph/), which examines the open reading frame of all genes and the promoter region of well‐annotated genes.

Polymorphic genes were then cross‐referenced to a list of 421 genes annotated by the microarray software Mapman 2.1.1 (Thimm *et al*., 2004) as being involved in biotic stress, and with genes shown to be significantly up‐ or down‐regulated in response to a PAMP, the bacterial flagellin protein flg22 (supplementary data in Zipfel *et al*., 2004). This approach focused on genes that may alter expression levels during an attack by pathogens, and therefore may contribute to partial resistance, but did not check every gene within each QTL interval. In addition, therefore, all the genes within the QTL regions were viewed as annotated loci on the TAIR website to see whether further genes should be included, based on their function reported elsewhere as being related to a resistance or stress response. A shortlist of candidates for partial resistance genes in the region of the major QTL, *QRps.JIC‐3.1,* is given in Table 2 and a list of possible candidates at the three minor QTLs in Table S2 (see Supporting Information).

**Table 2:** Shortlist of candidate genes within the regions of a major quantitative trait locus (QTL) in the Columbia × Landsberg (Col × Ler) recombinant inbred line (RIL) population, *QRps.JIC‐3.1*, which controls a large proportion of partial resistance to the virulent bacterial pathogen *Pseudomonas syringae* pv. *maculicola* ES4326, with annotations from The Arabidopsis Information Resource (TAIR) database. The numbers of polymorphisms between loci from Columbia and Landsberg *erecta* are as shown on the Polymorph GBrowse viewer. Bold type: genes reported to be involved in biotic stress. Italic type: genes reported to be involved in pathogen‐associated molecular pattern (PAMP) recognition

Chromosome	Locus	Description	Polymorphisms
3	AT3G04120.1	GAPC. Involved in glycolytic pathway, but also interacts with H_2_O_2_, potentially placing it in a signalling cascade induced by reactive oxygen species (ROS)	1
***3***	***AT3G04210***	***Disease resistance protein. TIR‐NBS domain. Induced by flg22***	***2***
**3**	**AT3G04220**	**TIR‐NBS‐LRR structure, suggestive of disease resistance protein**	**1**
3	AT3G04580.1	EIN4: ethylene receptor, subfamily 2. Has serine kinase activity	0
*3*	*AT3G04640.1*	*Glycine‐rich protein*	*1*

LRR, leucine‐rich repeat; NBS, nucleotide‐binding site; TIR, Toll/interleukin‐1 receptor.

**Figure 6:**
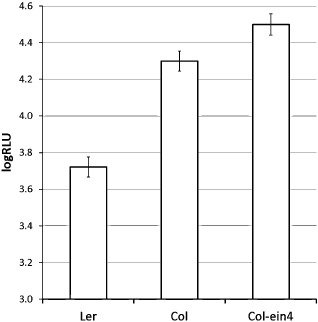
Mean disease scores on Landsberg *erecta* (Ler), Columbia (Col) and an *ein4* knockout mutant of Columbia expressed as log_10_ relative light units of luminescence emitted by the lux tag (logRLU) in *Pseudomonas syringae* pv. *maculicola* ES4326 inoculated by spraying at 5 × 10^7^ colony‐forming units (cfu)/mL. Bars indicate one standard error above and below the predicted mean. Least significant difference (*P <* 0.05) = 0.17.

One of the genes within the reduced set of 62 linked to the QTL *QRps.JIC‐3.1* on chromosome 3 was *EIN4*, one of five membrane‐bound ethylene receptors which negatively regulate the ethylene response pathway in the absence of ethylene. An *ein4‐1* mutant line derived from Col (Roman *et al*., 1995) was more susceptible to *Psm* than either Col or Ler (Fig. 6). The *EIN4* gene was sequenced from Col‐0 and Ler‐4, the parental accessions used to make the RIL population reported in this article. The coding sequences of *EIN4* were identical, and the only polymorphism identified in the promoter region by GBrowse Viewer was a single nucleotide polymorphism distant from the coding region, a transition from C to T at 337 bp from the start codon.

## Discussion

Four QTLs controlling partial resistance to a virulent isolate of *Psm*, ES4326, were identified in the *Arabidopsis* Col × Ler RIL population. Together, they accounted for almost 70% of the genetic variation in this population, whereas a major QTL on chromosome 3, named *QRps.JIC‐3.1*, explained 50% of the variation (Table 1). The analysis of partial resistance was greatly facilitated by the use of a lux‐transformed isolate sprayed onto *Arabidopsis* plants, which permitted tests to be performed on the large number of plants required for quantitative genetic analysis. Bacterial growth resulting from inoculation by spraying and by infiltration was highly correlated (Fig. 3). RILs are useful in biometrical genetics of *Arabidopsis* and other plants, because they allow tests to be replicated and lines to be replicated within tests, thus controlling for random variation in environmental conditions between tests and for random variation between plants within a test.

In another study of partial resistance to *Pseudomonas*, F2 progeny of *Arabidopsis* accessions Col × San Feliu were inoculated by dipping plants in a suspension of *P. syringae* pv. *tomato* (*Pst*) DC3000, such that the bacteria had to penetrate the leaves to cause infection, as in the spraying method used here. One major QTL on chromosome 5 was detected, explaining 77% of the variation in partial resistance, as well as two minor QTLs on chromosomes 1 and 4 (Kover and Cheverud, 2007). In a RIL population of Bayreuth × Shahdara, inoculated by infiltrating leaves with a suspension of *Pst* DC3000, two major QTLs were detected on chromosomes 2 and 5, and two minor QTLs on chromosomes 3 and 5 (Perchepied *et al*., 2006). QTLs with comparatively minor effects on nonhost resistance to *P. syringae* pv. *phaseolicola* were detected on chromosome 1 in the cross Ws‐3 × Nd‐1 and on chromosome 4 in Col‐5 × Nd‐1 (Forsyth *et al*., 2010). None of the QTLs for responses to *Pseudomonas* reported previously are at the same loci as those reported here, whereas the QTLs on chromosome 5 described by Kover and Cheverud (2007) and by Perchepied *et al*. (2006) were not in areas that are closely linked to *QRps.JIC‐5.1*. The support interval for the QTL on chromosome 1 reported by Forsyth *et al*. (2010) spanned most of the chromosome, so it is not possible to determine its relation with *QRps.JIC‐1.1*. Including the QTLs reported here, a total of four QTLs accounting for substantial fractions of partial resistance to *P. syringae* in *Arabidopsis* have been reported, in addition to several mapped, minor QTLs distributed throughout the genome and an unknown number of genes with small effects.

QTL analysis has been used in research on the relationship of defence pathways to resistance to *P. syringae* in *Arabidopsis*. A QTL on chromosome 4 regulating *PR‐1* expression induced by salicylic acid (SA) was associated with basal resistance to *P. syringae* in Bur‐0 (Ahmad *et al*., 2011), whereas QTLs on chromosomes 1, 3, 4 and 5 controlled SA‐induced resistance to *Pst* (Dobón *et al*., 2011). The QTL on chromosome 3 is intriguing, because it was located very close to *QRps.JIC‐3.1*, between *nga172* and *nga162* (Fig. 4B,C here and fig. S2 of Dobón *et al*., 2011); in contrast to *QRps.JIC‐3.1*, however, Col was more resistant than Ler, the QTL controlled a much smaller fraction of variation and resistance was not expressed in plants which had not been sprayed with SA. The linkage map of the Col × Ler population is not sufficiently dense to determine whether there is one gene in this region with contrasting effects on *P. syringae* pathovars *maculicola* and *tomato* or two linked genes.

The pattern of a few major QTLs, a larger number of minor QTLs and a substantial fraction of genetic variation which cannot be explained by identified QTLs is broadly similar to the results of other studies of partial resistance and other traits in plants, including *Arabidopsis*. The unmapped fraction of resistance is presumably controlled by genes with effects which are individually too small to be identified with confidence, falling below the threshold of statistical significance, but which collectively confer a substantial proportion of genetic variation. The presence of different genes in different sources of partial resistance is also broadly consistent with results on disease resistance in crop species (St Clair, 2010). The effect of major QTLs on partial resistance is reported to be more consistent than that of minor genes across environmental conditions (Kover and Cheverud, 2007; Perchepied *et al*., 2006). The major QTL reported here, *QRps.JIC‐3.1*, is therefore predicted to have a more environmentally stable effect than the three minor QTLs, with less genotype‐by‐environment interaction.

Diverse mechanisms contribute to partial resistance in crops (Stuthman *et al*., 2007) and the situation in *Arabidopsis* appears to be equally complex. They include, amongst others, pre‐formed barriers to infection, inhibition of developing haustoria, presence of pre‐formed antimicrobial compounds and loss of genes required for susceptibility. The modification of an ABC transporter which may be involved in leaf senescence confers partial resistance to rusts and powdery mildew of wheat (Krattinger *et al*., 2009), whereas basal resistance, in which plants respond to PAMPs (Boller and Felix, 2009), may have components in common with partial resistance. A search for genes involved in partial resistance should therefore take a comprehensive view and not be limited to homologues of genes which are already known to have a role in plant defence.

Fine mapping of the major QTL, *QRps.JIC‐3.1* on chromosome 3, enabled a substantial reduction from 205 candidate genes between flanking markers on a map calculated from the NASC markers alone to a region containing 62 candidate genes. The isolation of the gene controlling partial resistance to *Psm* in Col × Ler will require fine mapping in a much larger population to reduce the number of candidate genes still further, followed by experiments in which the effects on disease of Col and Ler alleles at each candidate locus are tested by, for example, transformation or gene knockout. Analysis of expression QTLs (Delker and Quint, 2011) might also refine the list of candidate genes. Several leading candidates at the major QTL were identified by bioinformatic analysis (Table 2), among which five are of particular interest. At3g04210, which is induced by the bacterial PAMP *flg22* (Zipfel *et al*., 2004), is highly expressed after treatment of leaves with SA (Tan *et al*., 2007). This gene is next to At3g04220, which responds to the necrosis and ethylene‐inducing peptide, Nep1‐Like Protein, from *Phytophthora parasitica* (Qutob *et al*., 2006). At3g04640 contributes to cell wall reinforcement and signal transduction of pathogen‐induced defences (Park *et al*., 2008). At3g04120 encodes GAPC, an isoform of glyceraldehyde‐3‐phosphate dehydrogenase (GAPDH). Co‐existence in the cytosol of GAPC and NP‐GAPDH, another isoform of GADPH, creates a bypass of carbon flux during glycolysis, which may increase the flexibility of responses to environmental stresses (Rius *et al*., 2008). The fifth gene in the list of leading candidates was *EIN4*, a sequence homologous to which was mapped to a QTL for resistance to Ascochyta blight in chickpea (Madrid *et al*., 2012). Although this gene contributed to partial resistance in a knockout experiment (Fig. 6), the only difference between the parents was a single nucleotide polymorphism in the promoter region, a considerable distance upstream from the transcription start site. The possibility that this polymorphism or other sequence variation in a still more distant part of the promoter could have affected the expression of *EIN4*, and thus the level of partial resistance, cannot be excluded.

Notable candidate genes identified at the other QTLs (Table S2) must be considered with caution because the support intervals span at least 150 genes in each case. They include a gene for a defensin‐like protein (a small, cysteine‐rich, antimicrobial peptide; Silverstein *et al*., 2005), one with a WRKY DNA‐binding domain and several involved in the ethylene response pathway. The QTL intervals included several possible *R* genes, identified by characteristic nucleotide‐binding site (NBS), leucine‐rich repeat (LRR) and Toll/interleukin‐1 receptor (TIR) domains (Bent, 1996), whereas the QTL on chromosome 5 overlapped with one of two ‘mega‐clusters’ of NBS‐LRR genes in Col (Holub, 2001). An estimated 1% of the *Arabidopsis* genome contains genes with features of *R* genes (Meyers *et al*., 2003), so the presence of such genes in QTL intervals here and in other crosses might simply reflect the widespread distribution of homologous sequences in the genome. Alternatively, partial resistance might be a weak form of gene‐for‐gene resistance (Li *et al*., 2006), but this has not been proven.

Any list of candidates for genes controlling partial resistance should not be limited, however, to those with a known function in disease, because susceptibility to pathogens involves many aspects of plant biology. For example, ERECTA, involved in the regulation of aerial plant development, affects resistance to bacterial wilt (Godiard *et al*., 2003), whereas AtTIP49a, essential for sexual organ development, interacts with RPM1 to suppress resistance (Holt *et al*., 2002). A combination of defence pathways which are integrated with plant growth and development, and can be regulated according to the type of invading pathogen, would provide a flexible, efficient defence system effective against diverse bio‐antagonists. The research reported here and by Perchepied *et al*. (2006), Kover and Cheverud (2007), Forsyth *et al*. (2010) and Dobón *et al*. (2011) provides a basis for the investigation of the possibly diverse mechanisms of partial resistance, making use of the genetic resources available in *Arabidopsis* as a model system.

## Experimental Procedures

### Plant lines

Ninety‐eight lines from the Ler‐0 × Col‐4 RIL population, plus Ler and Col, were donated by Clare Lister (John Innes Centre, Norwich, Norfolk, UK), and are available from NASC as the majority of Set 1 of 100 lines. Lines in Set 1 not included were N1906, N1984 and N1999, whereas an extra line, N1902, was added, giving a total of 98 lines (Lister and Dean, 1993). F1 crosses were made with lines N1934 and N1935. The *ein4‐1* mutant line (Roman *et al*., 1995), used for mapping the *EIN4* gene in a Columbia background, was a gift from Monica Pernas‐Ochoa (John Innes Centre) and is available from NASC (stock number N8053).

### Growth conditions and experimental design

Seeds were placed in sterile H_2_O at 4 °C for 2 days prior to sowing to break dormancy. For disease tests, seeds were sown in 8 : 1 peat‐based compost : grit in 5 × 5‐cm^2^ plastic pots, covered with a transparent lid. Each replicate experiment was arranged in a randomized incomplete block design with six blocks per experiment, with each line grown in five blocks. There were two replicates, giving a total of 10 plants per line. The parents, Ler and Col, were used as repeated controls with a total of 76 plants each. Trays of seedlings were placed in a growth room with a mixture of F70W/33 bulbs and Osram L58 W/77 Fluora bulbs giving approximately 100 μmol/m^2^/s light intensity on a 9‐h light/15‐h dark cycle at 24 °C. Lids were removed and seedlings were thinned out after 10 days to leave one plant per pot. Plants were inoculated when 5 weeks old.

### Bacterial strain and inoculation

A strain of *Psm* ES4326 transformed with the *luxCDABE* operon from *Photorhabdus luminescens* was provided by Dr Jun Fan (John Innes Centre) (Fan *et al*., 2008). Bacteria grown overnight at 28 °C in King's B medium with 50 μg/mL rifampicin were subcultured and harvested by centrifugation (3000 ***g***), and then diluted in 1 mL of 10 mm MgCl_2_ to yield a bacterial suspension of 5 × 10^7^ colony‐forming units (cfu)/mL; 0.02% Silwet L‐77 was added to the inoculation solution as a surfactant. Plants were sprayed evenly with the trays turned regularly until they were all covered in inoculum. Mock inoculum of 10 mm MgCl_2_ plus 0.02% Silwet L‐77 was applied in the same way to several trays of plants, also with a randomized planting design. The trays were then covered for 24 h to maintain high humidity.

An infiltration method of inoculation (Fan *et al*., 2008) was used for disease tests of Col × Ler F1 plants to keep leaves intact after scoring, so that they could be used to confirm the progeny genotypes. Bacteria were grown overnight and prepared as before (minus Silwet L‐77) to give a suspension containing 10^5^ cfu/mL, which was pressure infiltrated into leaves using a needleless syringe; control plants were infiltrated with 10 mm MgCl_2_. Plants were then returned, uncovered, to the growth room.

### Scoring disease

With both methods of inoculation, pathogen infection was scored at 3 dai (Fan *et al*., 2008). Briefly, the aerial parts of whole plants were cut off just below the rosette and ground in 1 mL of 10 mm MgCl_2_; 200 μL of the resulting liquid was placed into a 1.5‐mL clear Eppendorf tube and positioned in a luminometer to measure luminescence emitted by the lux tag in the range 370–630 nm as RLU. Pathogen scores were subjected to a log_10_ transformation to normalize the distribution of statistical errors; the resulting variate is referred to as logRLU.

### QTL mapping

Analysis and mapping of QTLs were performed using the software MapQTL 5 (Van Ooijen, 2004). The data in the genotype (.loc) and mapping (.map) files are available as supplementary data in Singer *et al*. (2006) and at the NASC website: http://arabidopsis.info/new_ri_map.html. A Kruskal–Wallis (KW) rank test was used to identify candidate regions of the Col × Ler map linked to partial resistance. Interval Mapping (IM) was then used to test the genetic effect of QTLs postulated by the KW test, using the logarithm of the likelihood ratio (LOD). A permutation test (Churchill and Doerge, 1994) with 1000 replicates was used to determine that the *P <* 0.05 significance threshold for the identification of QTLs was a LOD score of 3.3. Markers closely linked to QTLs were selected as co‐factors for MQM mapping. The original QTLs identified by IM were then more precisely defined using automatic co‐factor selection to select large numbers of initial co‐factors to represent each linkage group, followed by backwards elimination to define those that contributed the largest amounts of variation, thereby narrowing the regions containing QTLs.

### Fine mapping

After identifying the region on chromosome 3 in which the most potent QTL was located, markers from the NASC database were added and new markers were designed to fill the gaps between existing markers. The Sequence Viewer tool http://www.arabidopsis.org/servlets/sv enabled the scanning of the sequence to identify single nucleotide polymorphisms between Columbia and Landsberg which were used to design new markers. Restriction endonucleases (REs) predicted to cut at the site of each polymorphism were identified with NEBcutter V2.0 (http://tools.neb.com/NEBcutter2/index.php) (Vincze *et al*., 2003). Primer3 was used to design primer pairs (Sigma, Gillingham, UK; Table S1, see Supporting Information) for these regions (http://frodo.wi.mit.edu).

### Genotyping

DNA was extracted from leaf discs (diameter, 1 cm) using a pH 7.5 Tris‐HCl buffer, followed by precipitation from isopropanol. Pellets were resuspended in 70% ethanol, dried at room temperature, resuspended in Tris‐EDTA (TE) buffer and stored at −20 °C. Primers used for genotyping by polymerase chain reaction (PCR) and details of the PCRs are given in Table S1. Before genotyping the RILs, Col and Ler DNA extracts were tested to verify that primers amplified fragments of the expected size and that predicted polymorphisms were present. To verify the identity of F1 plants, a PCR was performed using the GAPC primers; *Eco*RV digestion confirmed that the F1 plants had fragments of all the sizes present in each parent.

### Sequencing

Primers were prepared and used to amplify the coding region of the *EIN4* gene, which is located near the potent partial resistance QTL on chromosome 3. The purified PCR product was sequenced by standard methods using Big Dye version 3.1 by the John Innes Centre Genome Laboratory. Sequences were viewed using Gap4 version 4.7 software.

## Supporting information


**Table S1** Primer pairs used to provide new CAPS markers for the fine mapping of single nucleotide polymorphisms in the region of *QRps.JIC‐3.1*, a quantitative trait locus (QTL) for partial resistance to *Pseudomonas syringae* pv. *maculicola* ES4326 in the Col × Ler recombinant inbred population on chromosome 3.
**Table S2** List of candidate genes within the regions of three minor quantitative trait loci (QTLs) identified in the Col × Ler recombinant inbred line (RIL) population (*QRps.JIC‐1.1*, *QRps.JIC‐2.1* and *QRps.JIC‐5.1*), that contribute to partial resistance to the virulent bacterial pathogen *Pseudomonas syringae* pv. *maculicola* ES4326, with annotations from The Arabidopsis Information Resource (TAIR) database. The number of polymorphisms between loci from Columbia and Landsberg *erecta* are as shown on the Polymorph GBrowse viewer. Bold type: genes reported to be involved in biotic stress. Italic type: genes reported to be involved in pathogen‐associated molecular pattern (PAMP) recognition.
