# Supplementary material for: Quantitative trait loci for partial resistance to Pseudomonas syringae pv. maculicola in Arabidopsis thaliana
Source: Mol Plant Pathol. 2013 Jun 3;14(8):828–37. doi: 10.1111/mpp.12043 (PMC3902988; doi:10.1111/mpp.12043)
Supplement: Supplementary file 1 — Table S1 Primer pairs used to provide new CAPS markers for the fine mapping of single nucleotide polymorphisms in the region of QRps.JIC‐3.1, a quantitative trait locus (QTL) for partial resistance to Pseudomonas syringae pv. maculicola ES4326 in the Col × Ler recombinant inbred population on chromosome 3. Table S2 List of candidate genes within the regions of three minor quantitative trait loci (QTLs) identified in the Col × Ler recombinant inbred line (RIL) population (QRps.JIC‐1.1, QRps.JIC‐2.1 and QRps.JIC‐5.1), that contribute to partial resistance to the virulent bacterial pathogen Pseudomonas syringae pv. maculicola ES4326, with annotations from The Arabidopsis Information Resource (TAIR) database. The number of polymorphisms between loci from Columbia and Landsberg erecta are as shown on the Polymorph GBrowse viewer. Bold type: genes reported to be involved in biotic stress. Italic type: genes reported to be involved in pathogen‐associated molecular pattern (PAMP) recognition. [file MPP-14-828-s001.doc]

Jenni C. Rant, Lia S. Arraiano, Matthieu Chabannes and James K.M. Brown

Quantitative trait loci for partial resistance to *Pseudomonas syringae pv. maculicola ES4326* in *Arabidopsis thaliana*

**Supplementary Table S1**

| **Marker name** | **Physical position (bp)** | **Forward primer 5'-3'** | **Tm° ^(1)^** | **Reverse primer 5'-3'** | **Tm°** | **Restriction enzyme** | **Fragment length (bases)** | **PCR prog.** ^(2)^ |
| --- | --- | --- | --- | --- | --- | --- | --- | --- |
| SGCSNP3952 | 1011054 | CGAAGCAGAAGTAGAAGAAGCA | 62.6 | ACCACAACTTGATGCCATGT | 62.9 | *Bpl*I | 275 | 2 |
| GAPC | 1081299 | ACGGAAAGACATTCCAGTC | 59.1 | CTGTTATCGTTAGGATTCGG | 58.6 | *Eco*RV | 1450 | 1 |
| Sm110_82,1 | 1226359 | GCTCTCTGGTTCGAATACGC | 63.8 | GAGGTTCTCTGATCCGATGG | 63.6 | *Mse*I | 500 | 2 |
| Sm47_292,7 | 1265286 | CTCATTTTCTCCACCCATCG | 64.3 | CAAGAACCCACAGGTCCATT | 63.7 | *Mse*I | 600 | 2 |
| Perl0425900 | 1344657 | CGATGGAGCAATGAGAGACA | 64.1 | TACCCGATAAGCCAATGACA | 62.8 | *Ssp*I | 425 | 3 |
| Sm205_229,2 | 1907061 | AAAGCAACTCGCAGCTTCTC | 63.6 | TAGCCGGCGACGTAATTAAA | 64.0 | *Mse*I | 500 | 2 |
| RPM1 | 2226920 | CTCTCCAGTTTCCCAGCAAG | 63.8 | GCATTCAGAAAGAGATGACACC | 62.5 | *Psi*I | 675 | 3 |

**Table S1** Primer pairs used to provide new CAPS markers for fine mapping single nucleotide polymorphisms in the region of *QRps.JIC-3.1*, a QTL for partial resistance to *Pseudomonas syringae* pv *maculicola* ES4326 in the Col x Ler recombinant inbred population on chromosome 3.

^(1)^ Tm° Melting temperature.

^(2)^ PCR programmes. 1: 30cycles of 94°C for 60s, 60°C for 30s, 72°C for 120s; 2: 30 cycles of 94°C for 60s, 65°C for 30s, 72°C for 60s; 3: 36 cycles of 94°C for 60s, 60°C for 30s, 72°C for 60s; all: terminated with 72°C for 10m then 10°C indefinitely. PCR reactions used 20ng DNA in a total of 20µL reaction mix, including 1µL forward and reverse primers at a final concentration of 0.5µM, 2 units Taq polymerase, 2µL 10 x reaction buffer and 2µL dNTPs (10mM). Products of RE digestion were run on 2% TAE-agarose gels with a 100bp ladder for fragment size control.

Jenni C. Rant, Lia S. Arraiano, Matthieu Chabannes and James K.M. Brown

Quantitative trait loci for partial resistance to *Pseudomonas syringae pv. maculicola ES4326* in *Arabidopsis thaliana*

**Supplementary Table S2**

| **Chr** | **Locus** | **Description** | **Polymor-phisms** |
| --- | --- | --- | --- |
| 1 | AT1G24062.1 | Defensin-like (DEFL) family protein. | 2 |
| *1* | *AT1G24140.1* | *Matrixin family. InterPro domain. Peptidoglycan binding-like.* | *1* |
| *1* | *AT1G24150.1* | *Group I formin. Localized to cell junctions. Polymerizes actin. Binds profilin.* | *3* |
| 1 | AT1G24190.1 | Enhances AtERF7-mediated transcriptional repression. | 2 |
| 2 | AT2G46240.1 | BAG protein. Knockouts enhanced susceptibility to *Botrytis cinerea*. | 3 |
| 2 | AT2G46430.1 | Downstream component of HR/resistance signalling pathways. | 2 |
| 2 | AT2G46440.1 | Positive regulator of resistance to avirulent fungal pathogen. | 3 |
| 2 | AT2G46450.1 | Positive regulator of resistance to avirulent fungal pathogen. | 3 |
| *2* | *AT2G46500.1* | *Phosphatidylinositol 3- and 4-kinase family / ubiquitin family* | *1* |
| 5 | AT5G44030.1 | Confers resistance towards bacterial and fungal pathogens, independent of salicylic acid, ethylene and jasmonate signaling. | 1 |
| 5 | AT5G44210.1 | ERF (ethylene response factor) subfamily B-1 of ERF/AP2 transcription factor family (ATERF-9). | 1 |
| **5** | **AT5G44420** | **Putative antifungal protein, AFP4.** | **1** |
| **5** | **AT5G44510** | **TIR-NBS-LRR structure, suggestive of disease resistance protein.** | **3** |
| 5 | AT5G44790.1 | RAN1:ATP dependent copper transporter required for ethylene response pathway. | 1 |
| ***5*** | ***AT5G44870*** | ***Disease resistance protein. TIR-NBS-LRR domains. Induced by flg22*** | ***0*** |
| *5* | *AT5G44910* | *Disease resistance protein; TIR domain. Induced by flg22.* | *2* |
| **5** | **AT5G45050** | **Disease resistance protein-related. Contains WRKY DNA-binding domain, NB-ARC domain, Leucine Rich Repeat.** | **6** |
| **5** | **AT5G45060** | **TIR-NBS-LRR structure, suggestive of a disease resistance protein.** | **20** |
| **5** | **AT5G45070** | **TIR domain, suggestive of a disease resistance protein.** | **3** |
| **5** | **AT5G45080** | **Disease resistance protein-related. Weak similarity to disease resistance protein rps4-RLD.** | **2** |
| *5* | *AT5G45110* | *Regulatory protein. NPR1 paralogue.* | *1* |
| 5 | AT5G45190.1 | Important roles upon infection with Cauliflower mosaic virus. | 1 |
| **5** | **AT5G45200** | **TIR-NBS-LRR structure, suggestive of disease resistance protein** | **5** |
| **5** | **AT5G45210** | **TIR-NBS-LRR structure, suggestive of disease resistance protein** | **7** |
| 5 | AT5G45220.1 | TIR domain. Similar to resistance protein (TIR-NBS-LRR class). | 6 |
| **5** | **AT5G45230** | **TIR-NBS-LRR structure, suggestive of disease resistance protein** | **3** |
| **5** | **AT5G45240** | **TIR-NBS-LRR structure, suggestive of disease resistance protein.** | **3** |
| **5** | **AT5G45250** | **TIR-NBS-LRR structure, suggestive of disease resistance protein. Identical to RPS4.** | **2** |
| **5** | **AT5G45260** | **TIR-NBS-LRR structure, suggestive of disease resistance protein.** | **10** |
| *5* | *AT5G45280.1* | *Contains InterPro domain pectinacetylesterase* | *4* |

**Table S2** List of candidate genes within the regions of three minor QTL identified in the Col x Ler RIL population (*QRps.JIC-1.1*, *QRps.JIC-2.1* and *QRps.JIC-5.1*), that contribute to partial resistance to the virulent bacterial pathogen *Pseudomonas* *syringae* pv. *maculicola* ES4326, with annotations from the TAIR database. The number of polymorphisms between loci from Columbia and Landsberg *erecta* are as shown on the Polymorph GBrowse viewer. Bold type: genes reported to be involved in biotic stress. Italic type: genes reported to be involved in PAMP recognition.
